# La baisse de la densité osseuse au cours des maladies inflammatoires chroniques de l'intestin: prévalence et facteurs de risqué

**DOI:** 10.11604/pamj.2013.15.70.2859

**Published:** 2013-06-25

**Authors:** Aida Ben Slama Trabelsi, Faouzi Abdellaoui, Mehdi Ksiaa, Ahlem Souguir, Hela Zeglaoui, Mohamed Ben Rejeb, Ahlem Brahem, Salem Ajmi, Ali Jmaa

**Affiliations:** 1 Service de Gastro-entérologie et Hépatologie Sahloul Sousse, Tunisia; 2 Service de Rhumatologie Hopital Farhat Hached Sousse, Tunisia; 3 Service de Médecine communautaire et préventive Sahloul Sousse, Tunisia

**Keywords:** Maladie de Crohn, recto-colite hémorragique, densité minérale osseuse, ostéopénie, ostéoporose, Crohn disease, ulcerative colitis, bone mineral density, osteopenia, osteoporosis

## Abstract

**Introduction:**

La baisse de la densité minérale osseuse représente la principale manifestation osseuse décrite au cours des maladies inflammatoires chroniques de l'intestin. En Tunisie, très peu d’études ont rapportés sa prévalence et ses facteurs de risque. Le but de ce travail était de déterminer la prévalence de la perte osseuse au cours des maladies inflammatoires chroniques de l'intestin, et rechercher ses facteurs de risque.

**Méthodes:**

Patients et méthodes: étude ouverte transversale, réalisée de 2007 jusqu’à 2012.

**Résultats:**

146 cas étaient colligés, dont 105 avaient une maladie de Crohn (71,9%) et 41 avaient une rectocolite hémorragique (28,1%). Il s'agissait de 62 hommes et 84 femmes. L’âge moyen était de 33,18 ans. La perte osseuse était trouvée chez 85 patients (58,2%). Il s'agissait d'une ostéopénie dans 57 cas et d'ostéoporose dans 28 cas. Les facteurs de risque de perte osseuse étaient une activité physique limitée (p=0,013), un indice de masse corporel ‘20 kg/m^2^ (p=0,015), une maladie active (p=0,035), l’étendue de l'atteinte intestinale (p=0,006) et une dose cumulée de corticothérapie dépassant 4,5g de Prednisone (p=0,003).

**Conclusion:**

La déminéralisation osseuse est une complication fréquente mais non constante. Ceci justifie un dépistage précoce chez les patients à risque, qui pourront ainsi bénéficier d'un traitement substitutif.

## Introduction

La perte osseuse représente une complication extradigestive fréquente des maladies inflammatoires chroniques de l'intestin (MICI). Cette complication s'ajoute aux autres difficultés de prise en charge de cette pathologie survenant chez des sujets jeunes. La perte osseuse est certe fréquente (75% selon les plus grandes séries) [1], mais non constante au cours des MICI. Ainsi, la mesure de la densité osseuse, ne pourrait pas être systématique chez ce groupe de malades. De ce fait, il est indispensable de définir un groupe à haut risque de développer une baisse de la densité osseuse, chez qui, sa mesure doit être systématique. En Tunisie, très peu d’études avaient rapportés sa prévalence et ses facteurs de risque. Ainsi, l'objectif de ce travail était de déterminer la prévalence de la perte osseuse chez les patients suivis pour maladie inflammatoire chronique de l'intestin et rechercher les facteurs de risque incriminés dans la baisse de la densité minérale osseuse.

## Méthodes

Il s'agissait d'une étude ouverte transversale, réalisée durant la période s’étendant du Janvier 2007 jusqu’à Juin 2012. Ont été inclus tous les patients ayant une maladie inflammatoire chronique de l'intestin diagnostiquée sur un faisceau d'arguments cliniques, radiologiques, endoscopiques et histologiques et ayant bénéficié d'une ostéodensitométrie. La mesure de la densité minérale osseuse (DMO) par absorptiométrie biphotonique aux rayons X (DEXA) a été pratiquée au niveau de la colonne lombaire antéropostérieure et au niveau des cols fémoraux. La densité osseuse a été évaluée par le calcul de la masse osseuse exprimée en g/cm^2^ et le T-score exprimé en déviations standards (DS). Selon l′Organisation Mondiale de la Santé (OMS), l'ostéoporose est définie par une masse osseuse au-dessous de-2.5 déviations standards en T score, et l'ostéopénie entre-1 et-2.5 DS en T score. L'activité de la maladie a été évaluée par l'Indice de Best pour la maladie de crohn, et la classification de Montréal pour la rectocolite hémorragique. Le phénotype de la maladie a été déterminé selon la classification phénotypique de Montréal (2005).

**Analyse statistique:** Les données ont été saisies et analysées au moyen du logiciel SPSS version 18. La comparaison des moyennes a été effectuée au moyen du test t de Student, et la comparaison de pourcentages par le test du chi-deux de Pearson. Une analyse initiale uni-variée de toutes les variables puis une analyse multi-variée des facteurs de risque dépendants pour trouver les facteurs de risque indépendantsont été realisés. Dans tous les tests statistiques, le seuil de signification a été fixé à 0,05.

## Résultats

### Caractéristiques de la population

Notre population était composée de 146 patients dont 105 (71,9%) atteints d'une MC et 41 malades (28,1%) présentant une RCH. Il s'agissait de 84 femmes (57,5%) et 62 hommes (42,5%) (Sex-ratio=0,73), âgés en moyenne de 33,18 ± 12,5 ans. Quarante et un patients (28,1%) étaient tabagiques actifs avec une consommation moyenne de 16,5 PA (Paquets-Année). Un faible apport calcique quotidien était noté chez 39 patients (26,7%), et 120 patients avaient rapporté une activité physique réduite (82,2%). L'indice de masse corporelle (IMC) moyen de nos malades était de 24,2 Kg/m^2^ ± 4,2. Dans le groupe MC, 27,6% des patients (29/105) avaient une localisation iléo colique; une MC iléale était trouvée chez 39% des malades (41/105), colique pure dans 31,4% des cas (33/105), et une localisation gastrointestinale dans 1,9% des cas (2/105). Dans le groupe RCH, 8 malades (19,5%) avaient une pancolite. Au moment de réalisation de la DMO, selon l'indice de Best, 59% des patients porteurs de MC (62/105) présentaient une maladie inactive et 41% (43/105) une maladie active (15 poussée minime, 26 poussée modérée).

Pour la RCH, 87,8% des patients (36/41) présentaient une maladie inactive. Sur le plan biologique, un syndrome inflammatoire biologique était noté chez 26 patients (17,8%). Dix huit patients avaient un syndrome biologique de malabsorption (12,3%). Dans notre série, 134 de nos patients (91,8%) étaient traités par une corticothérapie générale. La durée moyenne de la corticothérapie générale était de 7,48 ± 4,87 mois. La dose cumulée moyenne de corticothérapie générale reçue par nos patients était de 5,96 ± 2,08 g de Prednisone ou équivalent avec des extrêmes 2,04 et 15,22 g.

### Données de la mésure de la DMO

Tous les patients avaient bénéficié d'une ostéodensitométrie. Celle-ci était réalisée après un délai moyen de 5 ans par rapport à la date du diagnostic positif de la MICI. La masse osseuse moyenne était de 1,05 g/cm^2^ ± 0,16 au niveau du site vertébral et de 0,96 g/cm^2^ ± 0,13 au niveau du site fémoral. La DMO était normale chez 61 patients (41,8%) et basse chez 85 patients (58,2%). Il s'agissait d'une ostéopénie dans 57 cas (39%) et d'ostéoporose dans 28 cas (19,2%). Chez les malades ayant une ostéopénie, la baisse de la DMO était située au site vertébral dans 49,12% des cas (28/57), au site fémoral dans 24,56% des cas (14/57) et aux deux sites à la fois dans 26,32% (15/57). Chez les malades avec ostéoporose, la baisse de la DMO était au site vertébral dans 53,57% des cas (15/28), au site fémoral dans 21,43% des cas (6/28) et aux deux sites à la fois dans 25% (7/28). Tous les patients présentant une baisse de la DMO au moment du diagnostic (n=85) avaient reçu une supplémentation vitaminocalcique. Les bisphosphonates étaient prescrit chez les malades ayant une ostéoporose. Aucun de nos patients ayant une perte osseuse n'avait présenté de complication fracturaire sous traitement et ce, après un suivi moyen de 2 ans.

### Facteurs de risque de perte osseuse

L’étude univariée avait montré une relation statistiquement significative entre la perte osseuse et un âge supérieur à 38 ans (0,032), une consommation tabagique active (0,049), un apport calcique faible (p=0,0001), une activité physique réduite (p=0,024), un indice de masse corporelle bas et surtout <20 kg/m^2^ (p=0,033), un syndrome inflammatoire biologique (p=0,003), l'ancienneté de la maladie de plus de 12 mois (0,049), l’étendue de la maladie (0,001), et l'activité de la maladie (0,016), une durée totale de corticothérapie de plus de 12 mois (p=0,02), et une dose cumulée de corticothérapie générale > 4,5 g de Prednisone (p=0,001) (Tableau 1). Parmi les facteurs de risque qui étaient significativement corrélés avec une perte osseuse en étude uni-variée, seuls cinq étaient retenus comme facteurs de risque indépendants de perte osseuse en étude multi-variée: une activité physique limitée (p=0,013), l'indice de masse corporel < 20 Kg/m^2^ (0,015), l'activité de la maladie (p=0,035), l’étendue de l'atteinte intestinale (p=0,006), et une dose cumulée de corticothérapie dépassant 4,5g de Prednisone (p=0,003) (Tableau 2).

**Tableau 1:** Les facteurs prédictifs de perte osseuse au cours des maladies inflammatoires chroniques de l'intestin en étude univariée

	Densité minérale osseuse Basse	Densité minérale osseuse Normale	p
Age >38ans(%)	68	32	0,032
Tabac (%)	78	22	0,049
Apport calcique faible (%)	89,7	10,3	0,0001
Activité physique limitée (%)	62,5	37,5	0,024
IMC moyen (Kg/m^2^)	23,5 ±4,3	25,07 ±3,89	0,033
MICI ancienne >12 mois (%)	71,3	28,7	0,049
MICI active (%)	87,5	12,5	0,016
MICI étendue (%)	90	10	0,001
Syndrome inflammatoire (%)	84,6	15,4	0,003
Durée Corticothérapie ≥ 12 mois(%)	77,8	22,2	0,02
Dose Corticothérapie ≥4,5g P (%)	73,1	26,9	0,001

**Tableau 2:** Les facteurs prédictifs de perte osseuse au cours des densités minérales osseuses en étude multivariée

Variable	OR brut	IC à 95%	p	OR ajusté	IC à 95%	p
Tabac	3,6	[3,44-10,23]	0,049	-	-	-
Apport calcique↓	9,97	[3,31-30,06]	0,0001	-	-	-
Activité physique↓	2,66	[1,11 - 6,37]	0,024	4,57	[1,3715,17]	0,013
IMC <20kg/m^2^	0,91	[0,38 - 0,88]	0,033	0,86	[0,770,97]	0,015
MICI ancienne	2,42	[1,2 - 5,27]	0,049	-	-	-
MICI active	2,42	[1,2 - 29,2]	0,016	2,52	[1,32-4,05]	0,035
M étendue	9,3	[2,3 - 42,3]	0,001	5,53	[1,6318,72]	0,006
SIB	4,97	[1,61-15,31]	0,003	-	-	-
Corticothérapie ≥1 an	2,95	[1,10 -7,90]	0,02	-	-	-
Corticothérapie cumulée ≥ 4,5g	3,35	[1,62-6,92]	0,001	2,54	[1,74-3,12]	0,003

## Discussion

Les manifestations osseuses des MICI ont, ces dernières années, attiré l'attention des praticiens qui prennent en charge ces patients souvent jeunes. La perte osseuse apparaît actuellement comme une complication importante et souvent silencieuse des maladies digestives, qui aggrave leur morbidité et altère davantage la qualité de vie des patients [2]. La fracture est la seule complication cliniquement importante de l′ostéoporose, et le risque fracturaire dans l'ostéoporose des patients avec MICI est clairement établi dans la littérature. La plupart des études montrent une augmentation modérée du risque fracturaire par rapport à la population générale [3, 4].

Plusieurs études dans la littérature se sont intéressées au profil densitométrique des patients souffrant de MICI. Il en ressort que la prévalence de la perte osseuse chez ces malades est très variable et pourrait arriver jusqu’à 75% [1, 5–12]. La prévalence de l'ostéopénie varie de 40 à 50% et celle de l'ostéoporose de 5 à 37% [5]. Deux études tunisiennes publiées ont rapportés des prévalence de la perte osseuse de 45% et de 59% [13, 14]. Cette variabilité des prévalences rapportées, pourrait etre expliquée par la grande hétérogénéité des caractéristiques des populations d’étude. La prévalence de la perte osseuse chez nos malades rejoint celle de la littérature; elle était de 58,2% dont 39% ostéopénie et 19% ostéoporose.

Plusieurs facteurs sont incriminés dans la baisse de la densité osseuse. Certains facteurs sont liés au terrain. L'IMC étant le facteur le plus rapporté par les études dont notre étude [1, 7, 13–15]. La dénutrition au cours des MICI pourrait etre en rapport avec une carence d'apport qui se voit en cas de troubles dyspeptiques, de douleurs abdominales et de sténoses digestives, une malabsorption qui intéresse les apports protéino-énergétiques mais aussi vitaminiques et se voit en cas d'atteinte de l'intestin grêle ou en cas de résection intestinale, et l'entéropathie exsudative qui intervient dans la carence protéinique.

Bien que non confirmé dans des études randomisées, l′exercice physique est largement préconisé pour la prévention des fractures. Ainsi, l'exercice parait prévenir l′ostéoporose en augmentant la densité minérale osseuse, en réduisant la perte osseuse liée à l′âge, et par la restauration de l'os déjà perdu chez les personnes âgées. Il a été demontré dans une étude anglaise contrôlée randomisée en double aveugle portant sur 117 MC, que l'activité physique réduite est un facteur indépendant de perte osseuse au cours des MICI [16]. Dans notre série, l'activité physique réduite était correlé à la perte osseuse (p=0,013).

D'autres facteurs liés au terrain tel que l'age [1, 9, 13], le sexe [8, 13], le tabagisme [17, 18], et l'apport calcique quotidien [1, 13], ont été recherché, mais les résultats étaient discordants. Dans notre travail nous n'avons pas retrouvé de relation entre ces facteurs et la perte osseuse.

Les phénomènes inflammatoires sont également incriminés dans cette perte osseuse. Cette hypothèse est retenue devant la corrélation entre l'activité de la maladie et la densité minérale osseuse et l'implication des prostaglandines et de certaines cytokines pro inflammatoires comme l'IL1, l'IL6 ou le TNF ' dont l'expression est augmentée dans les MICI et qui ont un rôle clé dans le métabolisme osseux, en favorisant la résorption osseuse. La plupart des études de la littérature dont la notre [8, 14], ont démontré que l'activité clinique ou biologique sont des critères prédictifs de perte osseuse au cours des MICI.

L'impact de l’étendue de la MICI sur le profil densitométrique du patient n'a pas été largement évalué dans la littérature et peu d’études trouvés qui s'y intéressés [15, 19]. Dans l’étude de Robinson et al il n'a pas été trouvé de corrélation positive entre l’étendue de la MC ou la RCH, et le profil densitométrique [16]. Dans notre étude, une corrélation significative positive a été trouvée entre l’étendue de l'atteinte digestive et la baisse de la DMO en étude univariée (p=0,001), et en étude multivariée (0,006). La relation entre la durée d’évolution de la maladie et la DMO au cours des MICI est bien établie dans la littérature [6, 13]. Ceci n'a pas été retrouvé dans notre étude. Le type de MICI ne semble pas influencer la perte osseuse, La plupart des études, convergent vers l'absence de différence statistiquement significative entre les DMO des malades atteints de RCH de celles des patients ayant une MC [1, 8, 9]. De même, pour le siège et la forme de la maladie, le nombre de poussée, et la notion de resection intestinale.

Les corticoïdes contribuent à la perte osseuse par plusieurs mécanismes: d'une part ils diminuent la formation osseuse par inhibition des ostéoblastes, induction de leur apoptose et inhibition des facteurs de croissance et d'autre part ils accélèrent la résorption osseuse. La dose cumulée reçue de corticoïdes, et la durée du traitement ont été mis en cause selon les études [14, 16, 18, 19].

Semeao et al ont mis en évidence dans une étude réalisée chez 119 jeunes adultes avec MC, qu'une dose cumulée supérieure à 5g, une dose journalière supérieure à 7,5 mg, ou une durée d'exposition supérieure à 12 mois augmentaient de façon significative le risque de déminéralisation osseuse [19]. Cependant, l'influence de la corticothérapie sur la DMO n'est pas retrouvée dans toutes les études [7, 8]. Dans notre étude, seule une dose cumulée > 4,5g était retenue comme facteur de risque indépendant de perte osseuse.

La prise en charge de l'ostéoporose au cours des MICI repose sur une hygiène de vie consistant en la pratique d'une activité physique régulière, l’éviction du tabac et de l'alcool, la prévention des chutes, et un apport vitaminocalcique approprié. Une mesure de la densité osseuse s'impose dés que le diagnostic de MICI est fait chez un malade à risque ou lorsqu'une corticothérapie est envisagée. La prescription des bisphosphonates est destinée aux sujets ayant une ostéoporose confirmée.

## Conclusion

Au terme de notre étude et une revue de la littérature, nous pouvons conclure que la perte osseuse est une complication fréquente mais pas constante au cours des MICI; ainsi et devant un risque fracturaire modéré, la recherche de cette complication ne peut pas être de routine chez ces malades. Le dépistage par densitométrie osseuse, doit être systématique chez les patients à risque ayant: une activité physique réduite, un indice de masse corporelle < 20 Kg/m^2^, une maladie chronique active, une maladie étendue, et une dose cumulée de corticothérapie > 4,5g.
